# Characterization of the proteome of stable and unstable carotid atherosclerotic plaques using data-independent acquisition mass spectrometry

**DOI:** 10.1186/s12967-023-04723-1

**Published:** 2024-03-07

**Authors:** Zhichao Lai, Chaonan Wang, Xiaoyan Liu, Haidan Sun, Zhengguang Guo, Jiang Shao, Kang Li, Junye Chen, Jiaxian Wang, Xiangling Lei, Keqiang Shu, Yuyao Feng, Deqiang Kong, Wei Sun, Bao Liu

**Affiliations:** 1 Department of Vascular Surgery, Chinese Academy of Medical Science, Peking Union Medical College Hospital, Peking Union Medical College, Shuaifuyuan 1St, Dongcheng District, Beijing, 100730 People’s Republic of China; 2 Department of Hemangiomas & Vascular Malformations, Plastic Surgery Hospital, Chinese Academy of Medical Science, Peking Union Medical College, Beijing, China; 3 Proteomics Research Center, Core Facility of Instruments, Institute of Basic Medical Sciences Chinese Academy of Medical Sciences, School of Basic Medicine Peking, Union Medical College, Dongdansantiao 9St, Dongcheng District, Beijing, 100730 People’s Republic of China; 4 State Key Laboratory of Medical Molecular Biology, Institute of Basic Medical Sciences, Chinese Academy of Medical Sciences, Department of Pathophysiology, Peking Union Medical College, Beijing, China; 5 Eight-Year Program of Clinical Medicine, Chinese Academy of Medical Sciences, Peking Union Medical College Hospital, Peking Union Medical College, Beijing, China

**Keywords:** Carotid artery stenosis, Unstable carotid plaque, Proteomic, Ferroptosis

## Abstract

**Background:**

Currently, noninvasive imaging techniques and circulating biomarkers are still insufficient to accurately assess carotid plaque stability, and an in-depth understanding of the molecular mechanisms that contribute to plaque instability is still lacking.

**Methods:**

We established a clinical study cohort containing 182 patients with carotid artery stenosis. After screening, 39 stable and 49 unstable plaques were included in the discovery group, and quantitative proteomics analysis based on data independent acquisition was performed for these plaque samples. Additionally, 35 plaques were included in the validation group to validate the proteomics results by immunohistochemistry analysis.

**Results:**

A total of 397 differentially expressed proteins were identified in stable and unstable plaques. These proteins are primarily involved in ferroptosis and lipid metabolism-related functions and pathways. Plaque validation results showed that ferroptosis- and lipid metabolism-related proteins had different expression trends in stable plaques versus unstable fibrous cap regions and lipid core regions. Ferroptosis- and lipid metabolism-related mechanisms in plaque stability were discussed.

**Conclusions:**

Our results may provide a valuable strategy for revealing the mechanisms affecting plaque stability and will facilitate the discovery of specific biomarkers to broaden the therapeutic scope.

**Supplementary Information:**

The online version contains supplementary material available at 10.1186/s12967-023-04723-1.

## Introduction

Atherosclerosis (AS) is a slowly progressive chronic disease, but the rupture of plaques can lead to a series of acute clinical events. These events include myocardial infarction or stroke, which are major causes of morbidity and mortality in patients [1, 2]. In 2019, approximately 15 million people worldwide died because of ischemic heart disease and stroke, and these deaths accounted for 27.2% of all global deaths [3]. Among other things, unstable plaques increase the risk of ischemic stroke, regardless of the degree of carotid stenosis [4]. In recent years, the concept of plaque vulnerability has evolved, and progress has been made in predicting the risk of acute cerebrovascular events. While advances in noninvasive imaging techniques have facilitated the ability to identify unstable plaques through imaging, carotid imaging still has limitations [5–7]. To improve the early identification of plaque vulnerability, several studies have attempted to determine carotid plaque stability through the use of circulating biomarkers. However, none of the biomarkers have shown sufficient sensitivity and specificity to be confirmed in clinical practice [8, 9].

An in-depth understanding of the molecular mechanisms responsible for plaque instability can help identify biomarkers that represent unstable plaques. The progression of AS plaques from stable to unstable involves endothelial dysfunction, increased secretion of matrix metalloproteinases (MMPs), accumulation of lipids and inflammatory cells, and remodeling of the extracellular matrix (ECM). These pathological processes, together with hemodynamic factors, lead to fibrous cap rupture, tissue factor exposure, arterial thrombosis, and clinical events [10, 11]. In advanced AS, dysfunction-induced cell death leads to the formation of a characteristic necrotic core and vulnerable plaques. Current treatment of AS focuses on slowing disease progression and maintaining plaque stability. Unstable plaques are directly related to plaque rupture. Thus, biomarkers that can differentiate between stable and unstable plaques before serious complications from ruptured plaques arise may save patients from future cardiovascular and cerebrovascular events.

Several current studies using carotid plaque tissue have identified numerous molecular pathways implicated in the development of AS [12, 13]. However, genomic and transcriptomic changes do not fully reflect the true functional aspect. In recent years, proteomics has been developing rapidly in various fields, mainly based on the development of mass spectrometry technology and algorithms. Thus, numerous studies have attained many useful results in searching for potential drug targets and potential biomarkers of diseases and demonstrating the effectiveness of proteome-driven precision medicine [14–17]. Proteomic studies of AS plaques hold great promise for finding biomarkers associated with molecular biological mechanisms of AS.

In this study, data independent acquisition (DIA)-based quantitative proteomic analysis of stable and unstable plaques sampled by carotid endarterectomy (CEA) allowed us to determine the protein profiles of stable and unstable plaques and to identify new proteins that influence plaque instability and play important roles in plaque genesis and development. This may help to understand the molecular mechanisms of plaque instability, broaden the therapeutic spectrum, and provide a basis for identifying ideal biomarkers and new therapeutic targets for preventing future ischemic strokes.

## Materials and methods

### Optimization strategy of the carotid artery stenosis study and subject enrollment

The Optimization Strategy of Carotid Artery Stenosis (COAS-CAS) (Clinical trial: NCT05629000) is a prospective study to collect atherosclerotic carotid plaques and clinical characteristics (including routine blood tests and liver and kidney function tests) and imaging results from patients who underwent CEA at Peking Union Medical College Hospital (PUMCH). The study was approved by the ethics committee of PUMCH (No. JS-2966), and all methods were performed according to approved ethical guidelines. Written informed consent was obtained from all patients.

From May 2015 to March 2022, patients who underwent standard CEA or reverse CEA (eversion, eCEA) at PUMCH with asymptomatic carotid stenosis of 70% to 99% or symptomatic carotid stenosis of 50% to 99% (NASCET criteria) were consecutively enrolled in the study. The clinical data of all subjects was recorded at admission, and all patients underwent preoperative carotid duplex ultrasound scans with ≥ 50% carotid stenosis. Clinical data and test parameters for all individuals are shown in Additional file 1: Table S1. The practices and protocols in this study adhered to the principles of the Declaration of Helsinki.

### Human carotid endarterectomy samples

Samples of carotid plaque were obtained during surgery. Intraoperatively, complete excision was ensured, and the samples were gently rinsed with saline to remove blood and other impurities. Then, they were immediately fixed with 10% formalin and decalcified with 10% acetic acid. The plaque was split into proximal and distal regions and was centered at the carotid bifurcation and along the transverse axis of the artery. The region utilized to produce wax blocks was in the proximal section, which had a center that extended 20 mm in that direction, and the distal part, which had a center that extended 15 mm in that direction (if this range could not contain the entire plaque, the range was extended until the plaque was completely covered).

Plaques were classified as stable or unstable according to the following pathohistologic features: active inflammation; thin cap with large lipid core; endothelial denudation with superficial platelet aggregation; fissured plaque; intraplaque hemorrhage; stenosis > 90% [18]. Plaque classification was done performed independently by two pathologists with reference to American Heart Association (AHA) criteria [19, 20].

### Sample preparation

The tissue sections were scraped into EP tubes with a scalpel. Then, 20 µl of protein extract solvent was added to the sample. The samples were further incubated in a 100 °C water bath for 20 min and intermittently shaken. Protein samples were digested with the filter-aided sample preparation (FASP) method. The protein samples were reduced with 20 mM DTT for 5 min at 95 ℃ and then carboxyamidomethylated with 50 mM IAA at room temperature in the dark for 45 min. Then, the sample was loaded onto a 30 kDa ultracentrifugation filter, washed twice with UA buffer (containing 7 M urea and 50 mM Tris), and washed twice with 25 mM NH4HCO3. The treated samples were digested with trypsin (2 μg per 100 μg protein) in 25 mM NH4HCO3. The samples were digested at 37 °C overnight. The digested peptides were eluted from the 30 kDa filter, and the samples were desalted on C18 columns (3 cc, 60 mg, Oasis, Waters Corporation, Milford, MA). The desalted peptides were lyophilized by vacuum centrifugation and stored at − 80 °C.

### LC‒MS analysis of tissue extracts

All samples were analyzed on the UltiMate3000 system (Thermo Fisher Scientific) using an integrated monolithic C18 capillary column (ID, 75 µm, Length, 50 cm, Uritech, Beijing) and maintained at a constant column temperature of 60 °C throughout the entire experiment. Separation was achieved with stepped linear solvent gradients, all performed at a fixed flow rate of 1.5 µl/min with durations of 25 min. The organic modifier content (acetonitrile acidified with 0.1% v/v formic acid) was first increased from 5 to 20% in 15.5 min and then increased from 20 to 30% in 5 min. Then, it was increased from 30 to 50% in 1 min and increased to 90% in 0.1 min. Next, it was maintained for 1.3 min. Finally, it was increased from 90 to 5% in 0.1 min and maintained for 2 min.

The mass spectrometer was operated in positive mode with the Orbitrap Exploris 480 MS (Thermo Fisher Scientific). DIA was performed using the following parameters. For the full scan, the resolution was set at 120,000 with a normalized AGC target of 300%. The maximum injection time was set to 50 ms, and the scan range was from 350 to 1200 m/z. For DIA scans, the resolution was set at 30,000 with a normalized AGC target of 200% and a maximum injection time of 50 ms. A normalized HCD collision energy of 30% was used.

### Spectral library generation

The results were then imported to Spectronaut Pulsar (version 14, Biognosys, Switzerland) software and searched against the human SwissProt database to generate a DIA library. A maximum of two missed cleavages for trypsin was used, and cysteine carbamidomethylation was set as a fixed modification. Methionine oxidation, lysine deamination and carbamylation (+ 43) were set as variable modifications. The parent and fragment ion mass tolerances were set to 10 ppm and 0.02 Da, respectively. The applied false discovery rate (FDR) cutoff was 0.01 at the protein level.

### Data analysis

All findings were filtered using a Q value cutoff of 0.01 (corresponding to an FDR of 1%) to exclude outliers. The intensity of proteins was computed by adding the intensities of their respective peptides. Proteins identified in over 80% of each group's samples were retained for further examination. The k-nearest neighbor approach was utilized to fill in missing values. Principal component analysis (PCA) was implemented utilizing the web-based data analysis platform MetaboAnalyst 5.0 (https://www.metaboanalyst.ca/). Nonparametric tests (Wilcoxon rank-sum test) were used to evaluate the significance of differences between groups. Differentially expressed proteins (DEPs) were those that had a fold change of more than 2 and a p value of less than 0.05.

### Bioinformatics analysis

A volcano plot was generated and PCA was performed using MetaboAnalyst 5.0. A cluster heatmap was constructed using the R package “ggplots”. All differential proteins were analyzed using Ingenuity Pathway Analysis (IPA) software (QIAGEN, Ingenuity Systems, Mountain View, CA). Proteins were matched to disease and function categories, typical pathways from the Ingenuity Knowledge Base and other databases, and then ranked by their P value.

The DEP information was uploaded to the STRING database and validated for protein‒protein interaction (PPI) network analysis, and a minimal interaction score of 0.4 was set. The biomolecular interaction networks are described within the software Cytoscape (version 3.7.1).

Pearson correlation coefficients were used to analyze the association between clinical characteristics and DEPs; associations were considered statistically significant when the absolute value of the Pearson correlation coefficient was > 0.4 and the p value was < 0.05. The results of the correlations were presented as heatmaps using the "pheatmap" R package.

### Immunohistochemistry (IHC)

For IHC analysis, formalin-fixed, paraffin-embedded CEA samples were utilized. Tissue sections were deparaffinized and rehydrated in xylene and graded ethanol and boiled in sodium citrate buffer (pH = 6.0) for 2 min, followed by the addition of 0.3% H2O2 for 15 min to block endogenous peroxidase activity. Fetal calf serum (2%) was incubated for 20 min. Antibodies were incubated at 4 °C overnight, and then polymers were treated with anti-mouse and anti-rabbit immunoglobulins for 1 h. Images were obtained under a microscope after counterstaining with Mayer's hematoxylin and dehydration. The stained tissues were scanned with a Pannoramic Desk Scanner (3DHistech, Hungary) and observed with Case Viewer (version 2.3, 3DHistech, Hungary). The density of positively stained cells in cross-sections of carotid plaques was quantified with Image-Pro Plus (Ver. 6.0, Media Cybernetics, USA).

Primary antibodies for IHC against solute carrier family 1, member 5 (SLC1A5) (rabbit polyclonal, 20350-1-AP), apoptosis-inducing factor 2 (AIFM2) (rabbit polyclonal, 20886–1-AP), BH3 interacting-domain death agonist (BID) (rabbit polyclonal, 10988-1-AP), dipeptidyl peptidase 4 (DPP4) (rabbit polyclonal, 29403-1-AP), transferrin receptor protein 1 (TFR1) (mouse monoclonal, 66180–1-Ig), transferrin (TF) (rabbit polyclonal, 17435-1-AP), glutamate–cysteine ligase catalytic (GCLC) (rabbit polyclonal, 12601–1-AP), cholesteryl ester transfer protein (CETP) (rabbit polyclonal, 13459-1-AP), and apolipoprotein A-V (APOA5) (rabbit polyclonal, 18019-1-AP) were purchased from Proteintech (China).

### Statistical analysis

Baseline demographic data are expressed as the mean ± SD for continuous variables and as the frequency for categorical variables. Significant differences between the two groups were tested using t tests, chi-square tests and Fisher's exact tests. Comparisons between two groups after IHC staining were performed using a two-tailed Student’s t test. Pearson correlation analysis was performed to assess the relationship between clinical characteristics and DEPs. P values < 0.05 were considered statistically significant. All statistical analyses were conducted using GraphPad Prism software (ver. 7.0, GraphPad, USA) or R software (https://www.r-project.org/).

## Results

### Clinical characteristics of carotid plaques

**Fig. 1:**
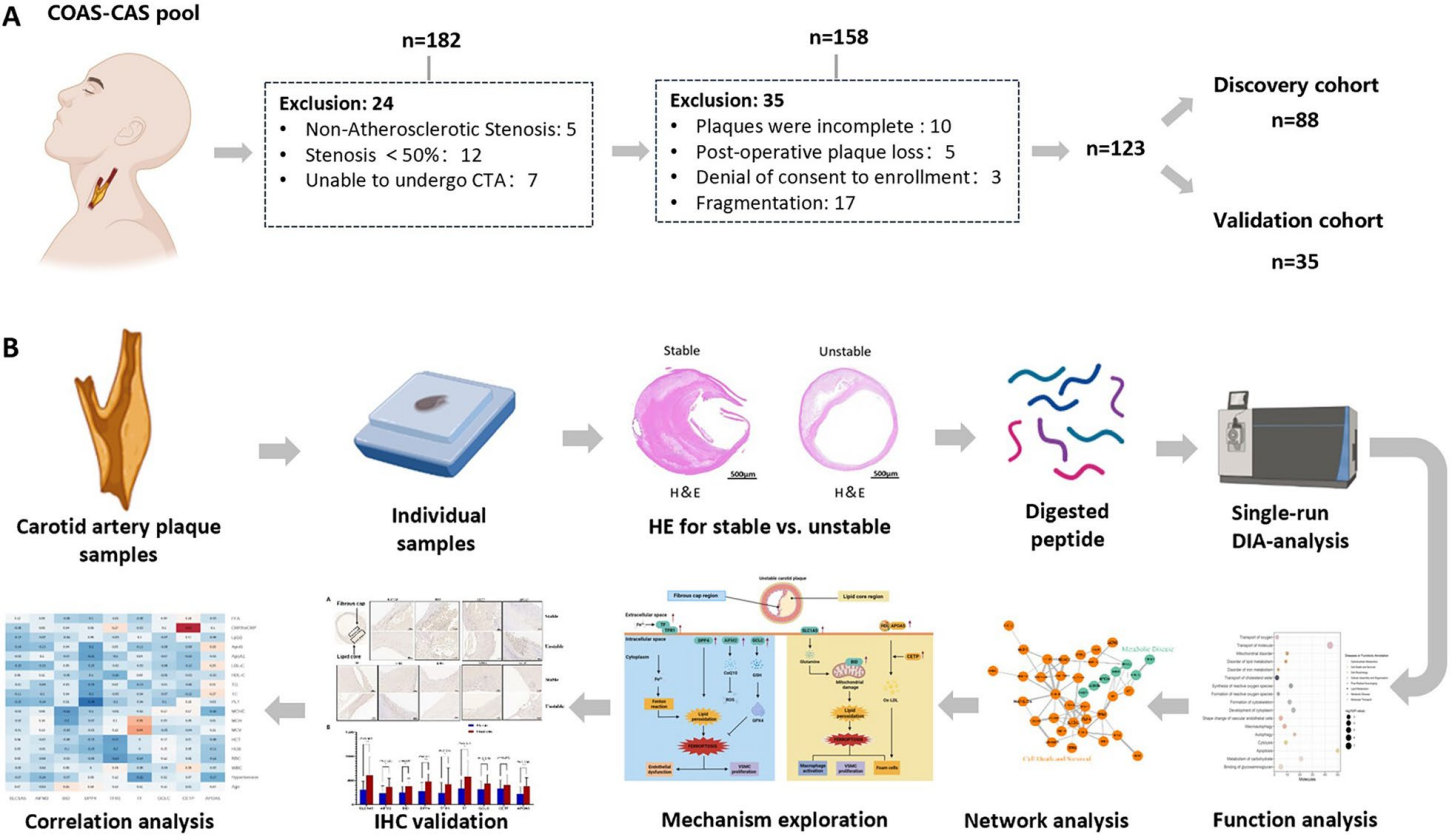
DIA-based proteomic analysis of carotid plaque cohorts. **A** Study cohort inclusion exclusion flowchart. **B** Overview of the carotid plaque proteomic workflow

A total of 182 subjects with carotid stenosis were recruited in this study between May 2015 and March 2022 at PUMCH. After the exclusion of 59 patients, 123 eligible patients were enrolled in the subsequent study cohort, of which 88 patients were used as the discovery group for proteomic analysis in DIA mode. A total of 35 patients were included in the validation cohort for IHC analysis. The study cohort inclusion and exclusion flowchart is shown in Fig. 1A.

The clinical baseline information of the enrolled patients is shown in Table 1 and Additional file 1: Table S1. The patients included 112 males and 23 females. The mean age was 66.53 ± 7.78 years, and the mean body weight was 72.82 ± 10.57 kg. The AS-related clinical indices (risk factors, AS-related diseases, degree of disease stenosis, and blood biochemical indicators of the severity of AS) were quantitatively and statistically analyzed for all enrolled patients. There was no statistically significant difference in clinical indicators, such as those of the routine blood and liver and kidney function tests, between the groups.

**Table 1:** Demographic and clinical characteristics of the subjects

	Discovery cohort (n=88)	Unstable (n=49)	*P *value	Validation cohort (n=35)	Unstable (n=15)	*P* value
	Stable (n=39)			Stable (n=20)		
Age (years)	65.73± 8.28	67.84±8.45	0.259^a^	65.25±7.36	66.07±3.69	0.697^a^
Weight (kg)	72.78±6.78	76.45±10.88	0.351^a^	69.55±6.66	0.069	0.452^a^
Gender (male, %)	86.486	80.000	0.824^b^	90.000	73.333	0.367^b^
Surgical side (%)			0.775^b^			0.176^b^
Left	43.243	46.667		60.000	33.333	
Right	56.756	53.333		40.000	66.667	
Degree of left carotid artery stenosis (%)			＜0.001^b^			0.644^b^
Mild	40.541	33.333		25.000	20.000	
Moderate	29.731	13.333		15.000	33.333	
Severe	27.027	42.222		60.000	46.667	
Occlusion	2.703	11.111		0.000	0.000	
Degree of right carotid artery stenosis (%)			0.319^b^			0.245^b^
Mild	24.324	28.889		35.000	13.333	
Moderate	13.514	11.111		5.000	20.000	
Severe	45.946	51.111		45.000	66.667	
Occlusion	16.216	8.889		15.000	0.000	
Hypertension (%)	72.973	64.444	0.283^b^	75.000	73.333	>0.999^b^
Diabetes (%)	37.838	48.889	0.151^b^	30.000	26.667	>0.999^b^
Hyperlipidemia (%)	35.135	24.444	0.121^b^	90.000	76.667	0.182^b^
Coronary heart disease (%)	27.027	24.444	0.745^b^	35.000	33.333	>0.999^b^
Smoking (%)	64.865	53.333	0.148^b^	35.000	46.667	0.321^b^
Cerebral infarction (%)	37.838	28.889	0.226^b^	35.000	33.333	>0.999^b^
Subclavian artery stenosis (%)	24.324	18.889	0.385^b^	20.000	20.000	>0.999^b^
Lower limb artery stenosis (%)	8.108	17.778	0.085^b^	10.000	6.667	>0.999^b^
Total cholesterol (mmol/L)	3.752	3.664	0.677	3.705	3.795	0.837
Triglycerides (mmol/L)	1.638	1.263	0.06	2.069	2.139	0.118
HDL-C (mmol/L)	0.994	1.008	0.773	0.963	1.083	0.248
LDL-C (mmol/L)	2.03	2.045	0.928	2.129	2.033	0.716
ApoA1 (g/L)	1.22	1.198	0.567	1.241	1.214	0.682
ApoB (g/L)	0.778	0.746	0.53	0.787	0.946	0.166
Lipoprotein(a) (mg/L)	205.8	212.5	0.909	159.1	312.5	0.094
hsCRP (mg/L)	2.779	3.502	0.575	1.92	2.894	0.340

^a^Two-sided p-value for numerical values were determined using unpaired multiple T-test

^b^Two-sided p-value for categories were calculated using Chi-square test and Fisher’s exact test. Data are presented as the mean ± standard deviation or number (%)

*P* value < 0.05 are indicated as significant

### Workflow

During the study period, 88 plaques in the discovery group were eventually enrolled for mass spectrometry analysis. In the validation group, IHC analysis was performed on 35 plaques from the final enrollment. During the discovery phase, we analyzed the proteome of CEA samples from 39 stable and 49 unstable plaques using the DIA method. First, we compared stable and unstable plaques to explore and identify proteins that are differentially expressed. In addition, we performed functional and pathway analyses of these DEPs and correlated the DEPs with clinical indicators, which were used to explore the molecular mechanisms of carotid plaques as well as the underlying mechanisms leading to plaque instability. Finally, DEPs were externally validated using IHC methods in the validation group (Fig. 1B).

### Protein identification in DIA-MS

A total of 6143 proteins were identified using a 1% false discovery rate (FDR), with an average of 3777 proteins detected in each sample and an average of 3736 and 3871 proteins detected in the stable and unstable groups, respectively (Additional file 1: Figure S1). During mass spectrometry, a total of 7 QC replicates were randomly arranged into the experimental samples. The technical variation of the experiment was assessed by calculating the coefficient of variation in protein abundance between the samples (Additional file 1: Figure S2) and showed good technical reproducibility.

### Quality assessment of carotid plaque samples

To avoid the effects of inconsistent sample collection on analysis and errors due to sample contamination, we assessed the quality of all samples before analysis. We used a previously reported quality marker panel to determine the degree of contamination of erythrocytes [21]. If the ratio differed more than two standard deviations from the mean of all samples within the cohort, the sample was flagged as a potentially contaminated sample and excluded from further analysis. Six samples in the cohort were found to show increased intensity of contamination markers and were therefore excluded from further analysis (Additional file 1: Figure S3). Additional file 1: Table S2 lists the proteins in each panel of quality markers.

### Differential proteomic analysis

**Fig. 2:**
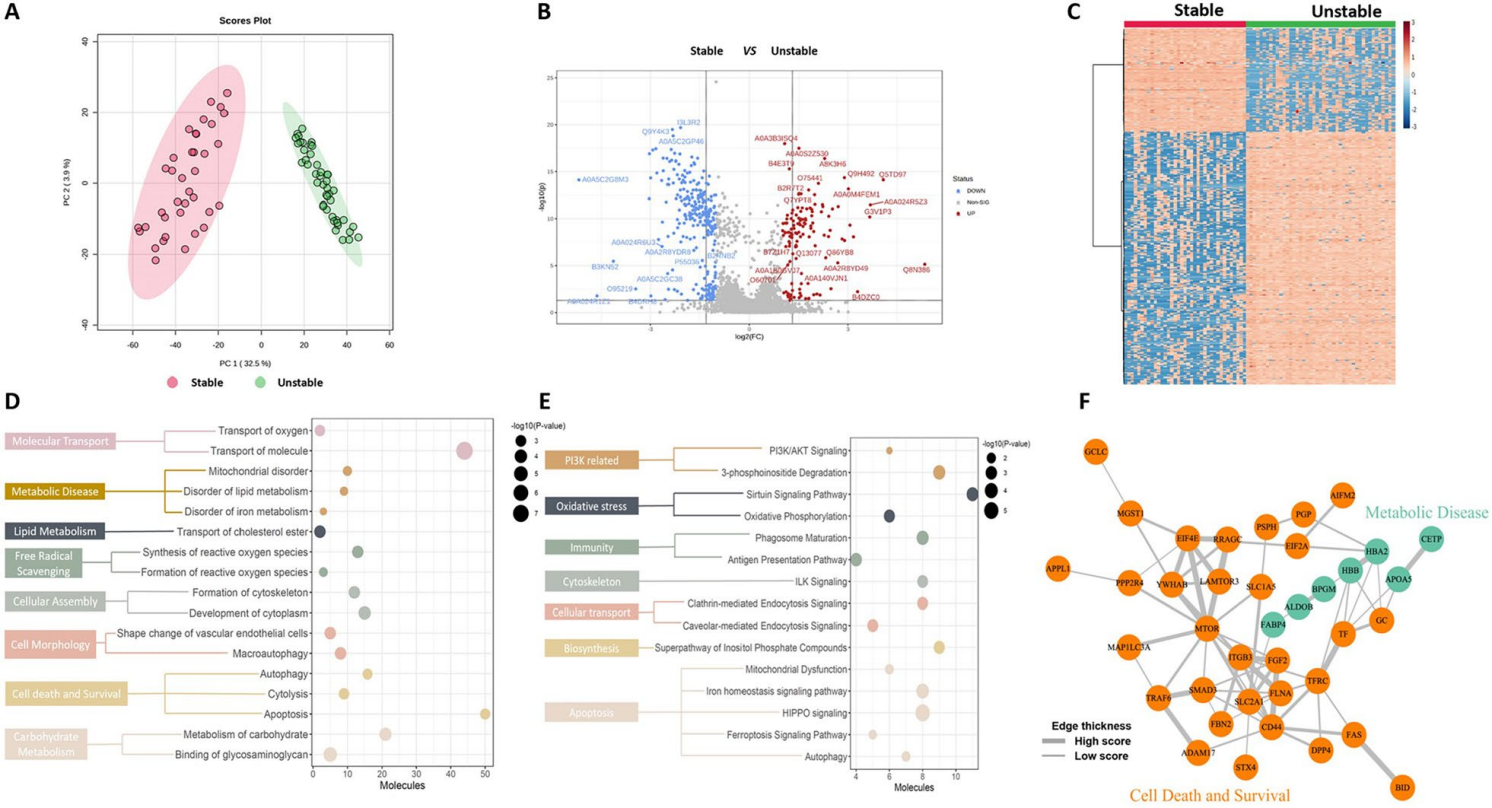
DEP analysis of the carotid plaque proteome. **A** PCA plots of stable compared with unstable samples by DIA analysis. **B** Volcano plots for stable compared to unstable samples by DIA analysis. **C** Heatmap of 397 DEPs between the 2 groups. **D** Ingenuity Pathway Analysis (IPA) function annotation analysis of the carotid plaque proteome. **E** IPA ingenuity canonical pathway analysis of DEPs. **F** The network of ferroptosis- and lipid metabolism-related DEPs. *DEPs* differentially expressed proteins. *PCA* principal component analysis

Unsupervised PCA of a total of 3999 common proteins in the two groups was performed to visualize the protein profiling differences among the stable and unstable plaques. The results showed that there was a significant difference between the stable and unstable plaque groups (Fig. 2A) (Additional file 1: Table S3). Next, DEPs between the unstable group and the stable group were identified as those with a standard fold change higher than 2 and a B-H adjusted P value of less than 0.05.

Thus, 397 proteins were identified, 123 of which were downregulated and 274 of which were upregulated (Fig. 2B) (Additional file 1: Table S4a). The DEP-based heatmap showed that the 2 groups expressed different proteomic patterns (Fig. 2C).

### Functional analysis of the DEPs

The DEPs of stable and unstable plaques were analyzed for canonical pathways and biofunctions using IPA. Analysis of disease and function showed that many DEPs involved in cellular assembly and organization, lipid metabolism, cell death and survival, free radical scavenging, metabolic disease, and cell morphology were activated (Additional file 1: Table S4b). Notably, cell death-related proteins were the most highly activated proteins, suggesting that stable plaques are closely associated with cell death during their evolution into unstable plaques (Fig. 2D). IPA of canonical pathways revealed enrichment of DEPs in the following pathways: cell death, cellular transport, immunity, biosynthesis, oxidative stress, cytoskeleton, intercellular junctions, and PI3K-related pathways (Additional file 1: Table S4c). Among them, the top canonical pathways were associated with cell death and PI3K (e.g., HIPPO signaling, iron homeostasis signaling pathway, PI3K/AKT signaling, and ferroptosis signaling pathway) (Fig. 2E).

**Fig. 3:**
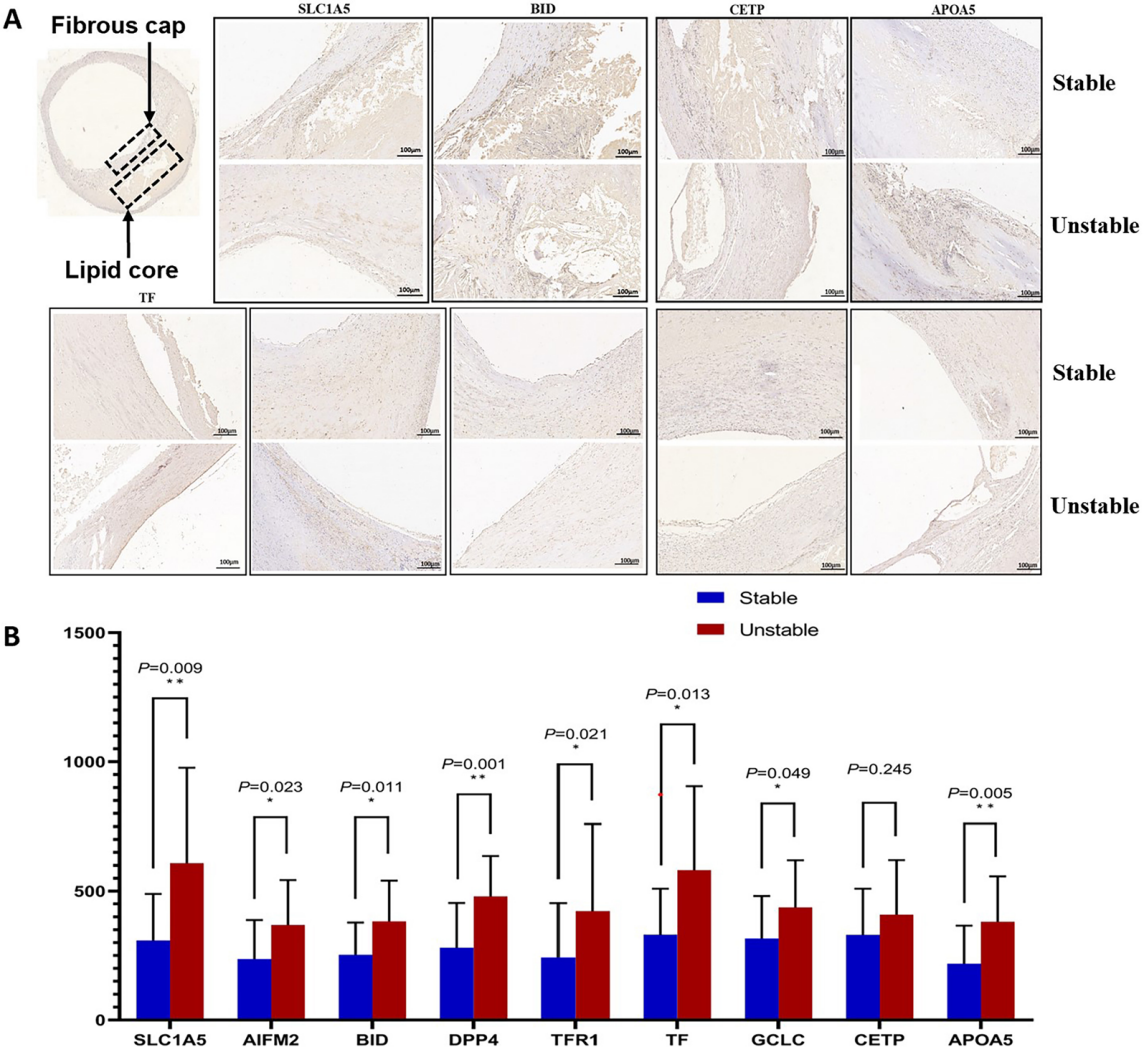
Validation of ferroptosis- and lipid metabolism-associated DEPs using IHC in a validation cohort. **A** Representative images (see Additional file 1: Figure S4a,b for 500 µm images) of immunohistochemical staining of TFR1, TF, AIFM2, DPP4, and GCLC proteins in stable and unstable plaques in the plaque fibrous cap region (black arrows) and immunohistochemical staining for SLC1A5, BID, and APOA5 proteins in the plaque lipid core region (black arrows). **B** In unstable plaques, the levels of TFR1, TF, AIFM2, DPP4, GCLC, SLC1A5, BID, and APOA5 were significantly increased, while other differences were not statistically significant. *IHC* immunohistochemistry. *TFR1* transferrin receptor protein 1; *TF* transferrin; *AIFM2* apoptosis-inducing factor 2; *DPP4* dipeptidyl peptidase 4; *GCLC* glutamate-cysteine ligase catalytic. *SLC1A5* solute carrier family 1, member 5; *BID* BH3 interacting-domain death agonist; APOA5, apolipoprotein A-V; *CETP* cholesteryl ester transfer protein. *P < 0.05; **P < 0.01 Student’s t test (two-tailed distribution)

The PPI network of DEPs was analyzed using the web application STRING and is depicted in Fig. 2F. In total, 52 nodes and 103 edges were identified. The DEPs were clustered into 2 clusters that may be involved in the functions of ferroptosis, lipid metabolism, transportation of lipoproteins, and oxidoreductase activity (Fig. 3F, Additional file 1: Table S5a-c).

The expression levels of ferroptosis-related proteins, including SLC1A5, AIFM2, BID, DPP4, TFR1, TF, and GCLC, were considerably higher in the group with unstable plaques, as shown by IPA and PPI network analysis of DEPs. In addition, the expression levels of lipid metabolism-, inflammation-, and autophagy-related proteins were dramatically altered. For example, APOA5, CETP, and CD44 were expressed at low levels in the stable plaque group and at high levels in the unstable plaque group, and MTOR showed the opposite trends.

### IHC validation in carotid plaques

Through bioinformatics analysis and results from the literature on plaque stabilization mechanisms, we selected validated proteins associated with ferroptosis and lipid metabolism. To understand where the differential proteins are expressed in plaques, we performed IHC analysis of 35 human carotid atherosclerotic plaques using another independent clinical cohort. IHC staining for identified proteins (SLC1A5, AIFM2, BID, DPP4, TFR1, TF, GCLC, APOA5, and CETP) (all upregulated) was performed on serial sections (Table 2) and was validated. The expression of TFR1, TF, AIFM2, DPP4, and GCLC proteins was most frequently observed in the fibrous cap region of stable plaques (Fig. 3) (Additional file 1: Figure S4a).

**Table 2:** The differently expressed proteins (DEPs) that were validated using immunohistochemistry (all up regulated) (unstable/stable)

Protein name	Accession	Gene symbol	FC (discovery)	FC (validation)	*P* –value (discovery)	*P-*value (validation)
Solute carrier family 1, member 5	Q15758	SLC1A5	2.76987	1.97175	1.67E-09	0.00948
Apoptosis-inducing factor 2	Q9BRQ8	AIFM2	2.06379	1.55662	0.00016	0.02345
Interacting-domain death agonist	A8ASI8	BID	5.40286	1.51099	5.47E-15	0.01129
Dipeptidyl peptidase 4	P27487	DPP4	2.46589	1.70935	2.55E-09	0.00178
Transferrin receptor protein Transferrin Glutamate--cysteine ligase catalytic	P02786 P02787 B4E2I4	TFR1 TF GCLC	2.29554 2.07101 2.51450	1.74235 1.75609 1.38121	2.58E-07 6.16E-06 2.95E-10	0.02189 0.01332 0.04928
Cholesteryl ester transfer protein Apolipoprotein A-V	A0A0S2Z3F6 A0A0B4RUS7	CETP APOA5	3.55635 2.19130	1.23646 1.74603	1.37E-09 1.36E-14	0.24549 0.00567

In contrast, most lipid core regions did not express the above proteins. Additionally, the expression levels of the above proteins were significantly increased in unstable plaques (P < 0.05). In the lipid core region of lipid-rich plaques, the protein expression levels of SLC1A5, BID and APOA5 were significantly higher in unstable plaques than in stable plaques (Fig. 3) (Additional file 1: Figure S4b). The exception was CETP, whose expression in the lipid core region of unstable plaques was not significantly higher than that in stable plaques (P = 0.245). This trend of increased expression was more pronounced in plaques with more intraplaque hemorrhage. The expression levels detected by IHC showed the same trend as those detected by DIA-proteomics.

### Correlation of DEPs and clinical characteristics

**Fig. 4:**
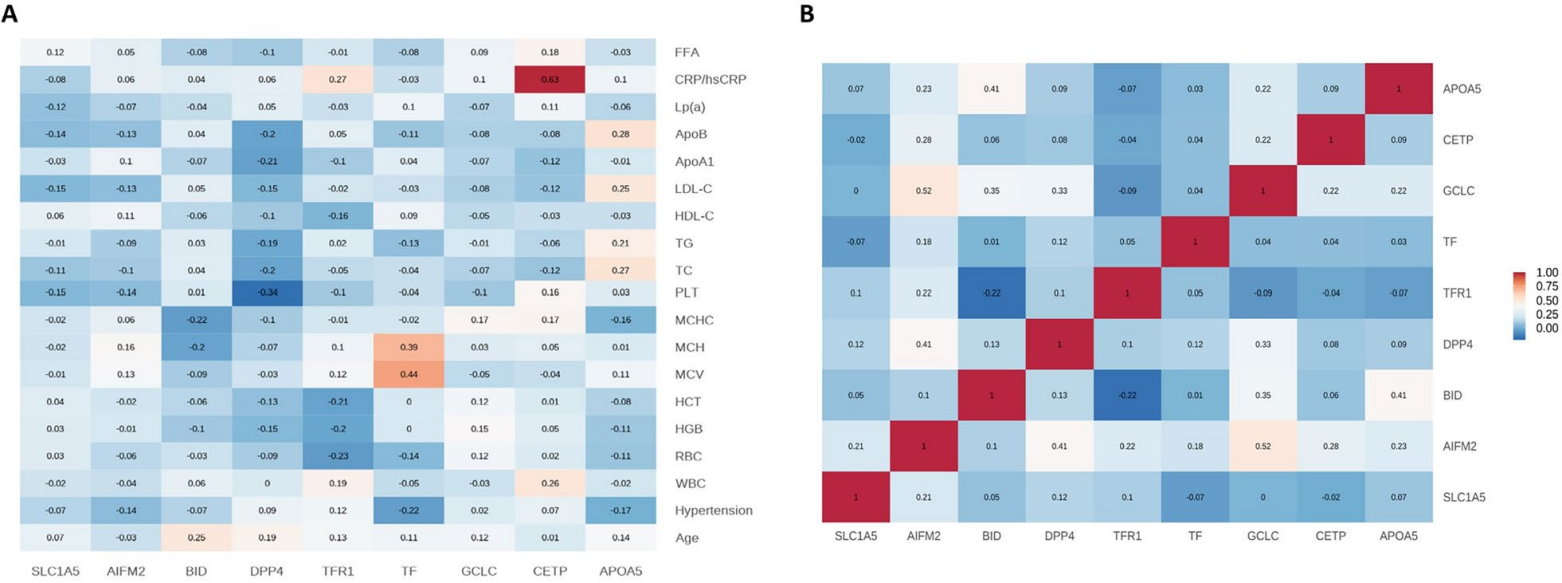
Pearson correlation analysis of DEPs and clinical characteristics. **A** Heatmap of the relationship between validated DEPs and clinical characteristics. **B** Hierarchical clustering based on validated DEPs. *DEPs* differentially expressed proteins

Pearson correlation analysis was performed to analyze the relationship between 9 DEPs, including SLC1A5, AIFM2, BID, DPP4, TFR1, TF, GCLC, CETP and APOA5, and the clinical data and test indicators, and AS-related clinical indicators were screened. Significant positive correlations were found between CETP and CRP (r = 0.63) and TF and MCV (r = 0.44). Our data may suggest that CETP is directly involved in the mechanisms associated with the ability of lipoproteins to alter the level of inflammation in the organism. In addition, according to the intrinsic correlations among the 9 DEPs, there was a positive correlation between GCLC and AIFM2 (r = 0.52), but the mechanism underlying this association needs to be further investigated (Fig. 4) (Additional file 1: Figure S5).

## Discussion

In this study, we compared stable and unstable carotid plaques. Based on the proteomic analysis of carotid plaques with IHC validation of relevant proteins and their correlations with clinical indicators, the processes of cell death, lipid metabolism, and inflammation were found to be associated with plaque stability. Our data reveal and reflect changes in carotid plaque proteomics. We demonstrated for the first time that changes in ferroptosis-related proteins are associated with unstable and ruptured carotid atherosclerotic plaques, and thus, they may be an independent factor of programmed cell death during AS lesions. We also found that lipid metabolism-related proteins correlate with inflammation-related clinical markers, which may increase our understanding of the molecular mechanisms of plaque instability. The study of the functions and mechanisms that lead to plaque instability will expand the scope of treatment and improve the prognosis of patients with AS.

Proteomic studies of AS plaques hold great promise for finding biomarkers associated with molecular biological mechanisms of AS. Therefore, the characterization of cellular proteins is of utmost importance to further understand plaque instability risk and new therapeutic pathways. Most early studies utilized 2D gel electrophoresis techniques for quantitative observation of proteins, but the number and types of proteins they could identify were very limited[22, 23]. Recently, the development of new proteomics tools, such as high-throughput mass spectrometry, has accelerated the discovery of new candidate markers and gradually revealed functional pathways in tissues or diseases. In recent years, there have been successive studies using liquid chromatography‒mass spectrometry (LC‒MS) and data-independent acquisition (DIA)-MS techniques for proteomic analysis of carotid plaque tissue. However, most of these studies had relatively small sample sizes and did not address the issue of interindividual variation in candidate biomarkers well [24–28]. To our knowledge, this is the first study to compare stable and unstable plaque lesions using DIA-MS techniques. To identify proteins involved in influencing atherosclerotic plaque stability, we used plaques from patients undergoing CEA. By quantitatively analyzing the composition of the plaque proteome and its variation in unstable plaques, we identified and characterized potential proteins and their new potential roles in unstable plaque tissue that affect plaque stability.

### Proteomic characterization of carotid plaques

By comparing the proteomes of stable and unstable plaques, we were able to identify numerous functions that were disturbed in unstable plaques, including cellular assembly and organization, lipid metabolism, cell death and survival free, cell morphology, metabolic disease and radical scavenging. These functions were activated in unstable plaques, shedding light on the mechanisms underlying the development of unstable plaques.

Previous studies have reported the presence of reverse cholesterol transport and AS-related mechanisms that promote cholesterol efflux from foam cells in ruptured carotid plaques. Studies have also identified the presence of apolipoproteins in carotid plaques from symptomatic patients and the upregulation of proteins associated with LDL particle clearance and macrophage-derived foam cell differentiation [12, 29]. To our knowledge, the distinguishing feature in unstable plaques is a high lipid load, especially an increase in the free cholesterol (FC)/cholesterol ester (CE) ratio. In the particular case of macrophages, higher FC levels are associated with the formation of foam cells [30, 31]. Additionally, low-density lipoprotein (LDL) undergoes further oxidation in plaques, and high concentrations of oxidized LDL (Ox-LDL) in plaques correlate with susceptibility to rupture of atherosclerotic lesions [32]. In the present study, lipid metabolism-related proteins, mainly involved in CE transport and lipid transport-related proteins (e.g., AKR1C1, APOA5, CES1, CETP, ITGB3, OSBPL2, SLC9A3R2, and STX12), were expressed at higher levels in unstable plaques than in stable plaques. Thus, the activation of lipid metabolism may be involved in the complex molecular mechanisms that affect plaque stability.

During atherosclerotic plaque development, cells within the plaque undergo constant turnover, and many vital factors mediate cell proliferation, recruitment, and programmed cell death. In this study, many DEP functions were associated with programmed cell death, involving cell lysis, apoptosis, autophagy, and ferroptosis. Previous studies have suggested that macrophages, smooth muscle cells (SMCs) and endothelial cells in plaques may undergo several types of programmed death, including apoptosis, autophagy, pyroptosis, and necrosis, but whether it is a protective or pathogenic mechanism remains controversial [33]. It has been suggested that macrophage accumulation and death may promote the formation and expansion of lipid necrosis cores and plaque instability [34]. Execution of different forms of cell death programs and the stages in which they occur may lead to different outcomes in plaques.

In addition, an important finding of this study was that the levels ferroptosis-related proteins (e.g., SLC1A5, BID, DPP4, TFR1, TF, AIFM2, and GCLC) were significantly different between the stable and unstable plaque groups, which has not been mentioned in previous studies.

Ferroptosis is a novel form of programmed cell death that is dependent on ROS production and iron overload. It occurs when the glutathione (GSH)-dependent lipid peroxidation repair system is compromised through lethal ROS accumulation, ultimately leading to oxidative cell death. It has been shown to be involved in the development and progression of AS through different signaling pathways, mainly including redox homeostasis, iron homeostasis, impaired mitochondrial activity and metabolism of amino acids, lipids and sugars [35–37]. Furthermore, ROS accumulation, lipid peroxidation, and iron deposition are important features of advanced atherosclerotic plaques. Previous studies have indicated that increased free iron also accelerates inflammation and foam cell formation [38], and significantly elevated levels of ferritin and TfR1 have been reported in carotid plaques from male patients with abnormal iron metabolism [28]. Loss of activity of GPX4, a key enzyme in ferroptosis, also leads to the accumulation of large amounts of lipid peroxides and ROS, further affecting the progression of AS. The overexpression of GPX4 reduced lipid peroxidation and inhibited the development of plaques in ApoE-/- mice [39]. In our study, IPA functional analysis showed that proteins related to oxygen free radical scavenging, iron metabolism disorders, lipid metabolism disorders, and mitochondrial dysfunction were upregulated in the unstable plaque group. Moreover, we found that HIPPO signaling, the iron homeostasis signaling pathway, mitochondrial dysfunction and the ferroptosis signaling pathway were significantly dysregulated in both groups in the pathway analysis. Other studies have reported that HIPPO signaling is involved in the progression of AS and has been shown to be involved in the activation of ferroptosis by modulating TFR1 to increase the intracellular Fe2 + concentration and induce intracellular ROS production [40, 41]. In our study, the above pathways were predicted to be more dysregulated in the unstable plaque group, suggesting that ferroptosis is more likely to occur in the unstable plaque group. This is possibly due to increased endothelial ROS triggered by peroxidation of LDL in endothelial cells, decreased nitric oxide (NO), inflammation in macrophages and foam cell formation, and iron-catalyzed free radical reactions. This leads not only to ferroptosis but also to the oxidation of LDL in endothelial cells, smooth muscle cells or macrophages, all of which are involved in influencing the formation of unstable plaques.

In this study, we also validated differentially expressed proteins associated with ferroptosis and lipid metabolism using an independent set of clinical cohorts to reveal the mechanisms behind their influence on plaque instability, which may provide a new direction of exploration. A total of 9 proteins (SLC1A5, AIFM2, BID, DPP4, TFR1, TF, GCLC, CETP, and APOA5) were validated. Among them, SLC1A5, BID, DPP4, TFR1, TF, CETP and APOA5 positively regulated ferroptosis and lipid metabolism, while AIFM2 and GCLC negatively regulated ferroptosis.

In the present study, we observed increased expression levels of SLC1A5, BID, CETP and APOA5 proteins in the plaque lipid core region. Previous studies have shown that the formation of a lipid necrotic core and thin fibrous cap is often present in unstable plaques along with macrophage infiltration, and apoptosis and secondary necrosis of foam cells and SMCs are thought to be important causes of necrotic core development [42]. SLC1A5 is a cell surface transporter that mediates the uptake of L-glutamine (Gln). Gln enters the cell via SLC1A5. It generates glutamate in the mitochondria and enhances aerobic phosphorylation of mitochondria. Additionally, Gln generates large amounts of reactive oxygen species that mediate the formation of lipid peroxidation and promote ferroptosis [39, 43]. BID is a member of the pro-apoptotic Bcl-2 protein family that is essential for death receptor-mediated apoptosis in many cell systems, and its transactivation of mitochondria can mediate the loss of mitochondrial integrity and dysfunction. Further studies have revealed that BID can transactivate mitochondria during ferroptosis, causing mitochondrial damage and linking ferroptosis and mitochondrial damage [44, 45].

According to our results, the expression levels of SLC1A5 and BID in the lipid core region were significantly higher in the unstable plaque group than in the stable plaque group, which has not been reportedmentioned in previous studies. Therefore, we speculate that SLC1A5 and BID may also be regulated in the process of lipid peroxidation. This process can cause macrophage activation and foam cell formation and eventually cause ferroptosis in macrophages and SMCs, thus affecting the stability of plaques. We also observed increased expression levels of both CETP and APOA5 in the plaque lipid core. However, our validation results showed that there was no significant difference in the expression of CETP between the unstable plaque group and the stable plaque group. In the correlation analysis of clinical indicators, we found a strong correlation between CETP and CRP. Additionally, in a previous study on CETP and cardiovascular risk, CETP plasma levels were found to be negatively correlated with high-sensitivity C-reactive protein [46], which as an inflammatory marker that indirectly reflects the level of inflammation in the body. Taken together, our data may suggest that CETP is important for the anti-inflammatory function of HDL, and its role in the development of AS requires further investigation.

**Fig. 5:**
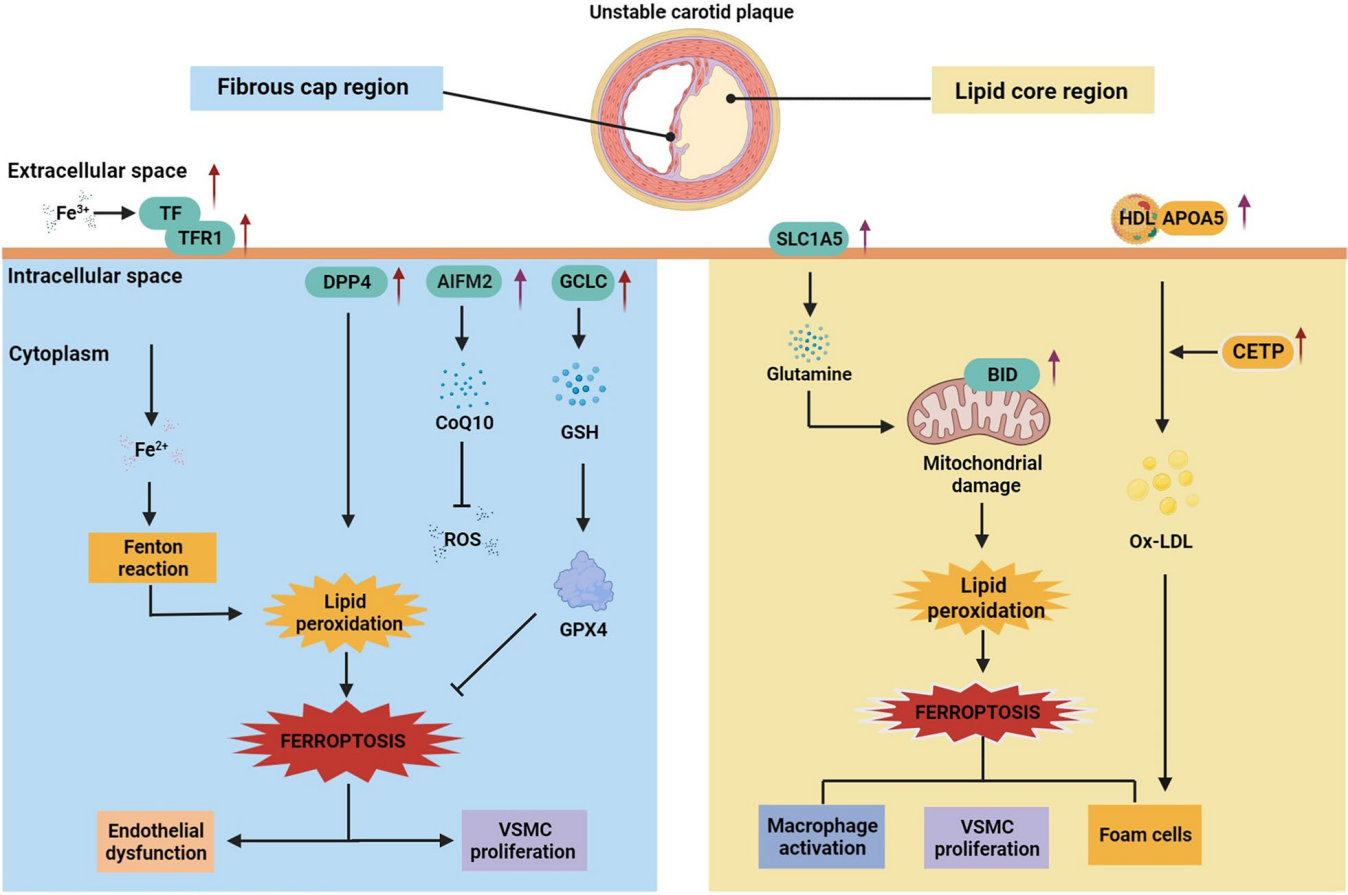
Hypothetical characterization of altered molecular mechanisms in cells in the fibrous cap and lipid core regions of unstable carotid plaques. The expression of key proteins of ferroptosis and lipid metabolism is significantly increased in patients with unstable plaques, and there are mechanisms associated with both the promotion and inhibition of iron death. This possibly indicates that cells in different regions of the plaque are regulated by key proteins of ferroptosis that subsequently lead to increased plaque instability and expansion of the necrotic core. *TFR1* transferrin receptor protein 1; *TF* transferrin; *AIFM2* apoptosis-inducing factor 2; *DPP4* dipeptidyl peptidase 4; *GCLC* glutamate-cysteine ligase catalytic; *SLC1A5* solute carrier family 1, member 5; *BID* BH3 interacting-domain death agonist; *APOA5* apolipoprotein A-V; *CETP* cholesteryl ester transfer protein; *GPX4* glutathione peroxidase 4; *GSH* glutathione; *CoQ10* coenzyme Q10; *ROS* reactive oxygen species; *HDL* high-density lipoprotein; *ox-LDL* oxidized low-density lipoprotein

In contrast, we observed increased expression levels of TFR1, TF, DPP4, AIFM2 and GCLC proteins in the fibrous cap region of the plaques, with significantly higher expression in the thin-fibrous cap region in the unstable plaque group than in the stable plaque group. The regulation of iron homeostasis is particularly important for the maintenance of overall redox homeostasis. Overloaded iron acts as a catalyst for redox reactions and can lead to oxidative stress-induced cytotoxicity [47]. Extracellular Fe3 + can bind to TF and be transported into the cell via TFR1, leading to an overload of the iron pool and the generation of ROS via the Fenton reaction. This leads to the accumulation of lipid peroxides, which is an important factor contributing to ferroptosis [48]. In this study, we found that TF and TFR1 were highly expressed in the thin-fibrous cap region of unstable carotid plaques; however, previous studies [49] have shown that TFR1 accumulates in large numbers mainly in the area of foam cell aggregates., and Wwe hypothesized that this discrepant finding was mainly due to the fact that the because the plaque fibrous cap was thinnest in the shoulder of the vessel wall tissue at the junction with the plaque, where smooth muscle cells are usually scarce or absent. This location, and is the most vulnerable rupture location., Itwhich also contains manya large number of activated macrophages and is, the location of the presence of iron transport phenomena. Moreover, it demonstrates, and a close association with iron homeostasis and inflammatory responses that accelerate plaque erosion or disruption.. Therefore, we suggest that ferroptosis is also present in the process of TF and that TFR1 affects plaque stability and accelerates plaque progression. Additionally, it could be an important ferroptosis-related target to prevent plaque progression. The current findings suggest that the pathways that antagonize ferroptosis are the glutathione-based GSH-GPX4 axis and the FSP1-CoQ10-NAD(P)H pathway [50]. Among them, AIFM2 (also known as ferroptosis inhibitory protein 1 FSP-1), a traditional inducer of apoptosis in mitochondria, is a potent ferroptosis resistance factor that protects cells from ferroptosis induced by GPX4 deficiency. And itIt was found that the anti-ferroptosisantiferroptotic function of FSP1-CoQ10-NAD(P)H was independent of glutathione levels in cells, and that this antioxidant system acts at the membrane level [51]. The GCLC has an independent, nonclassic role in preventing ferroptosis by maintaining glutamate homeostasis [52]. We also found high expression of AIFM2 and GCLC in the fibrous cap regions in unstable carotid plaques. It is evident that relatively independent ferroptosis antagonistic mechanisms may coexist in the process causing plaque instability (Fig. 5). In addition, we also found a significant correlation between GCLC and AIFM2. Although their pathways to inhibit ferroptosis are relatively independent, we speculate that there may be a possible regulatory role between them. However, this is pending further study. DPP4 is a serine protease that is widely expressed in many cell types, including endothelial cells, fibroblasts, and lymphocytes, and resides mainly as a dimer in cell membranes [53]. Many studies have shown that DPP4 can contribute to the process of endothelial dysfunction, inflammation, and AS, and despite strong preclinical evidence, several large-scale clinical trials have failed to demonstrate that it reduces the occurrence of major adverse cardiovascular events [54, 55]. Our findings revealed that DPP4 was highly expressed in the fibrous cap region of unstable plaques., and previous studies have shown that DPP4 is widely expressed in the endothelium of plaque neovasculature, and that the inhibition of DPP4 prevents the formation of foam cells and the release of pro-inflammatoryproinflammatory cytokines, which is one of the are important factors affecting plaque stability. In addition, DPP4 has been found to bind to NOX1, leading to the generation of ROS in the cell membrane and plasma, resulting in massive accumulation of intracellular lipid peroxides and ferroptosis [56]. Therefore, the results of this study provide a new direction for the specific mechanism by which DPP4 affects plaque stability, and itsDPP4 inhibitors may become a new therapeutic target.

## Conclusions

Through proteomic analysis, we found significant differences in protein expression patterns between stable and unstable carotid plaques. It is possible that the regulatory mechanisms of these proteins contribute to plaque instability or help protect cells near unstable plaques. In Fig. 5, we integrate the major findings of this study, describing a hypothetical molecular mechanistic model of unstable atherosclerotic plaques. A notable feature of the findings of this study is that we identified the possible presence of ferroptosis and changes in lipid metabolism among the mechanisms affecting plaque stability. Thus, ferroptosis may dominate the associated proteomic changes. Although this study is not necessarily conclusive per se, it adds to the relevant evidence that ferroptosis is involved as an independent factor in programmed cell death during atherosclerotic lesions. Additionally, the data provided in this study are expected to provide a good reference for future studies of the role of proteins in atherogenesis and plaque development.

Because only a small number of patients with advanced ankylosing spondylitis were recruited in this study, future validation using larger cohorts (especially multicenter cohorts) is warranted. In addition, the potential mechanisms of these proteins during plaque progression remain unclear. Thus, molecular biology and relevant animal models should be used to elucidate possible mechanisms affecting plaque progression and instability and improve our understanding of plaque instability.

### Supplementary Information

**Additional file 1: Fig S1.** Number of proteins identified and quantified with a 1% false-discovery rate (FDR) in each sample in the Stable and Unstable. **Fig S2.** Pearson correlation analysis of QC samples by DIA analysis. **Fig S3.** Histograms of log2 transformed ratios of the summed intensity of the proteins in the respective quality marker panel and the summed intensity of all proteins in discovery cohort. **Fig S4**. a-b. The validation of ferroptosis and lipid metabolism associated DEPs using IHC in an independent cohort. A, Representative images show the immunohistochemical staining of TFR1, TF, AIFM2, DPP4, and GCLC proteins in stable and unstable plaques in the plaque fibrous cap region. B, Immunohistochemical staining for SLC1A5, BID, and APOA5 proteins in the plaque lipid core region. **Fig S5.** a-b. Pearson correlation analysis of DEPs and clinical characteristics. A, The heatmap of the relationship between validated DEPs and clinical characteristics. B, The heatmap of the inner relationship of validated DEPs. DEPs, differentially expressed proteins. DEPs, differentially expressed proteins. **Table S1.** a Summary of demographics of discovery cohort. Table S1b Summary of demographics of validation cohort. Table S1c Clinical Features of All Patients. Table S1d Clinical Features of DIA-MS. Table S1e Clinical Features of Patients for Immunohistochemical Staining. **Table S2.** The panel that showed increased intensities of contamination markers. **Table S3.** Carotid plaque proteins quantified by DIA analysis. **Table S4.** Proteome differntial analysis of stable and unstable. Table S4a Differential proteins between stable and unstable by DIA analysis. Table S4b Enriched function of DEPs between stable and unstable. P value indicates the siginificances of pathways and functions. Z-score indicates the activation (positive value) or inhibition (negative value) status of functions. Table S4c Enriched pathway of DEPs between stable and unstable. P value indicates the siginificances of pathways and functions. Z-score indicates the activation (positive value) or inhibition (negative value) status of pathways. **Table S5.** PPI network analysis between DEPs. Table S5a Functional interaction analysis of PPI networks for DEPs. Table S5b PPI network analysis of ferroptosis-associated proteins. Table S5c PPI network analysis of lipid metabolism-associated proteins.

## Data Availability

The raw data supporting the conclusions of this article will be made available by the authors, without undue reservation. Requests to access these datasets should be directed to ZL and CW.

## Abbreviations

AIFM2: Apoptosis-inducing factor 2
APOA5: Apolipoprotein A-V
AS: Atherosclerosis
BID: BH3 interacting-domain death agonist
CEA: Carotid endarterectomy
CE: Cholesterol ester
CETP: Cholesteryl ester transfer protein
DEPs: Differentially expressed proteins
DPP4: Dipeptidyl peptidase 4
DIA: Data independent acquisition
ECM: Extracellular matrix
FC: Free cholesterol
FDR: False discovery rate
GCLC: Glutamate–cysteine ligase catalytic
Gln: l-glutamine
IHC: Immunohistochemistry
IPA: Ingenuity Pathway Analysis
LDL: Low-density lipoprotein
MMPs: Matrix metalloproteinases;
Ox-LDL: Oxidized LDL
PCA: Principal component analysis
PPI: Protein‒protein interaction
PUMCH: Peking Union Medical College Hospital
SLC1A5: Solute carrier family 1, member 5
SMC: Smooth muscle cells
TFR1: Transferrin receptor protein 1
TF: Transferrin
