# Supplementary material for: Characterization of the proteome of stable and unstable carotid atherosclerotic plaques using data-independent acquisition mass spectrometry
Source: J Transl Med. 2024 Mar 7;22:247. doi: 10.1186/s12967-023-04723-1 (PMC10921703; doi:10.1186/s12967-023-04723-1)
Supplement: Supplementary file 1 — Additional file 1: Fig S1. Number of proteins identified and quantified with a 1% false-discovery rate (FDR) in each sample in the Stable and Unstable. Fig S2. Pearson correlation analysis of QC samples by DIA analysis. Fig S3. Histograms of log2 transformed ratios of the summed intensity of the proteins in the respective quality marker panel and the summed intensity of all proteins in discovery cohort. Fig S4. a-b. The validation of ferroptosis and lipid metabolism associated DEPs using IHC in an independent cohort. A, Representative images show the immunohistochemical staining of TFR1, TF, AIFM2, DPP4, and GCLC proteins in stable and unstable plaques in the plaque fibrous cap region. B, Immunohistochemical staining for SLC1A5, BID, and APOA5 proteins in the plaque lipid core region. Fig S5. a-b. Pearson correlation analysis of DEPs and clinical characteristics. A, The heatmap of the relationship between validated DEPs and clinical characteristics. B, The heatmap of the inner relationship of validated DEPs. DEPs, differentially expressed proteins. DEPs, differentially expressed proteins. Table S1. a Summary of demographics of discovery cohort. Table S1b Summary of demographics of validation cohort. Table S1c Clinical Features of All Patients. Table S1d Clinical Features of DIA-MS. Table S1e Clinical Features of Patients for Immunohistochemical Staining. Table S2. The panel that showed increased intensities of contamination markers. Table S3. Carotid plaque proteins quantified by DIA analysis. Table S4. Proteome differntial analysis of stable and unstable. Table S4a Differential proteins between stable and unstable by DIA analysis. Table S4b Enriched function of DEPs between stable and unstable. P value indicates the siginificances of pathways and functions. Z-score indicates the activation (positive value) or inhibition (negative value) status of functions. Table S4c Enriched pathway of DEPs between stable and unstable. P value indicates the siginificances of pathw [file 12967_2023_4723_MOESM1_ESM.zip › ╕╜▒φ/Supplemental Figure.docx]

**Fig S1.** Number of proteins identified and quantified with a 1% false-discovery rate (FDR) in each sample in the Stable and Unstable.


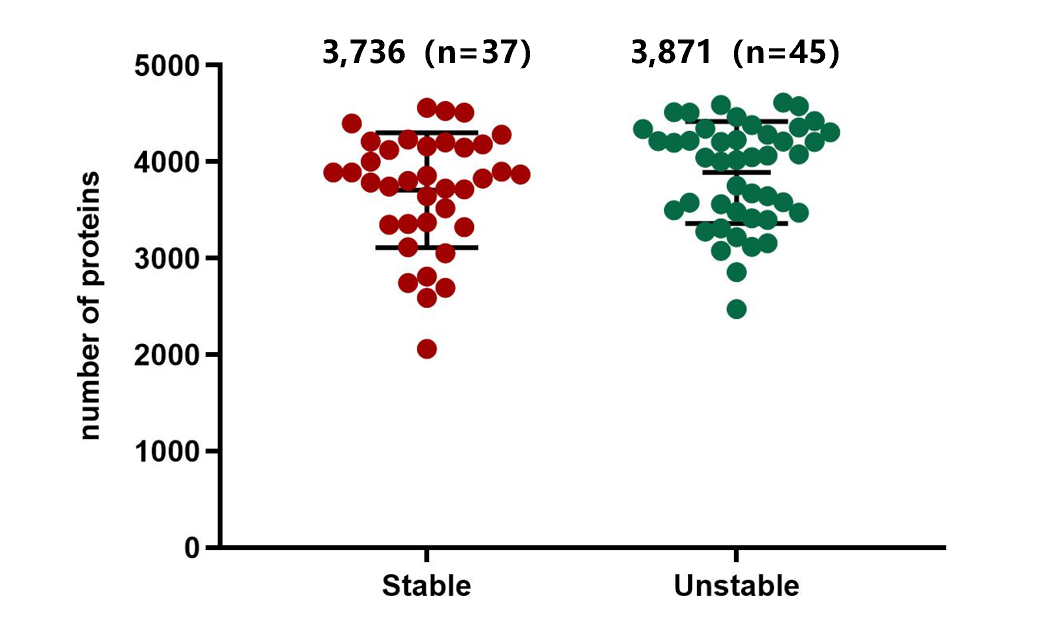


**Fig S2.** Pearson correlation analysis of QC samples by DIA analysis.


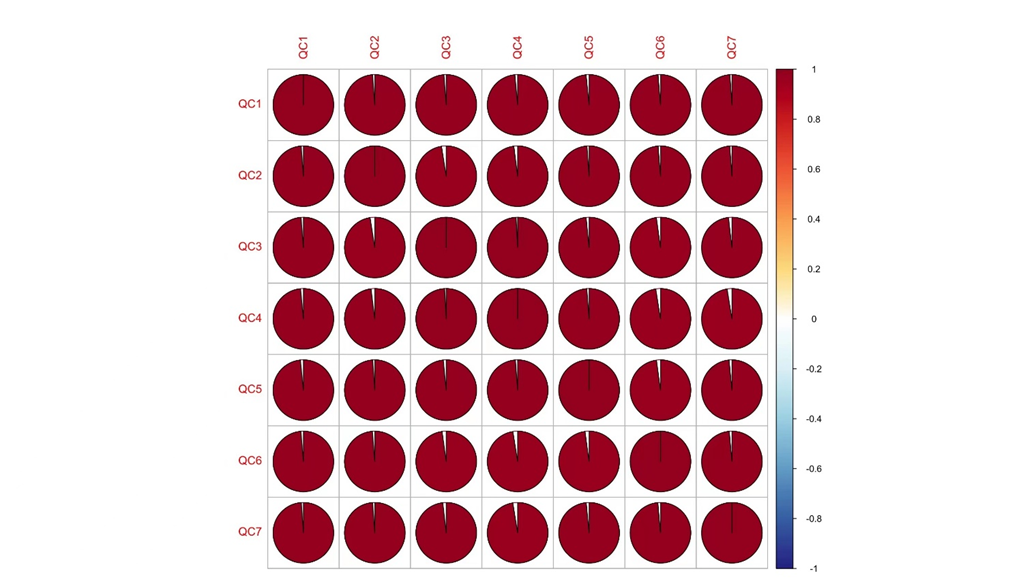


**Fig S3.** Histograms of log2 transformed ratios of the summed intensity of the proteins in the respective quality marker panel and the summed intensity of all proteins in

discovery cohort.
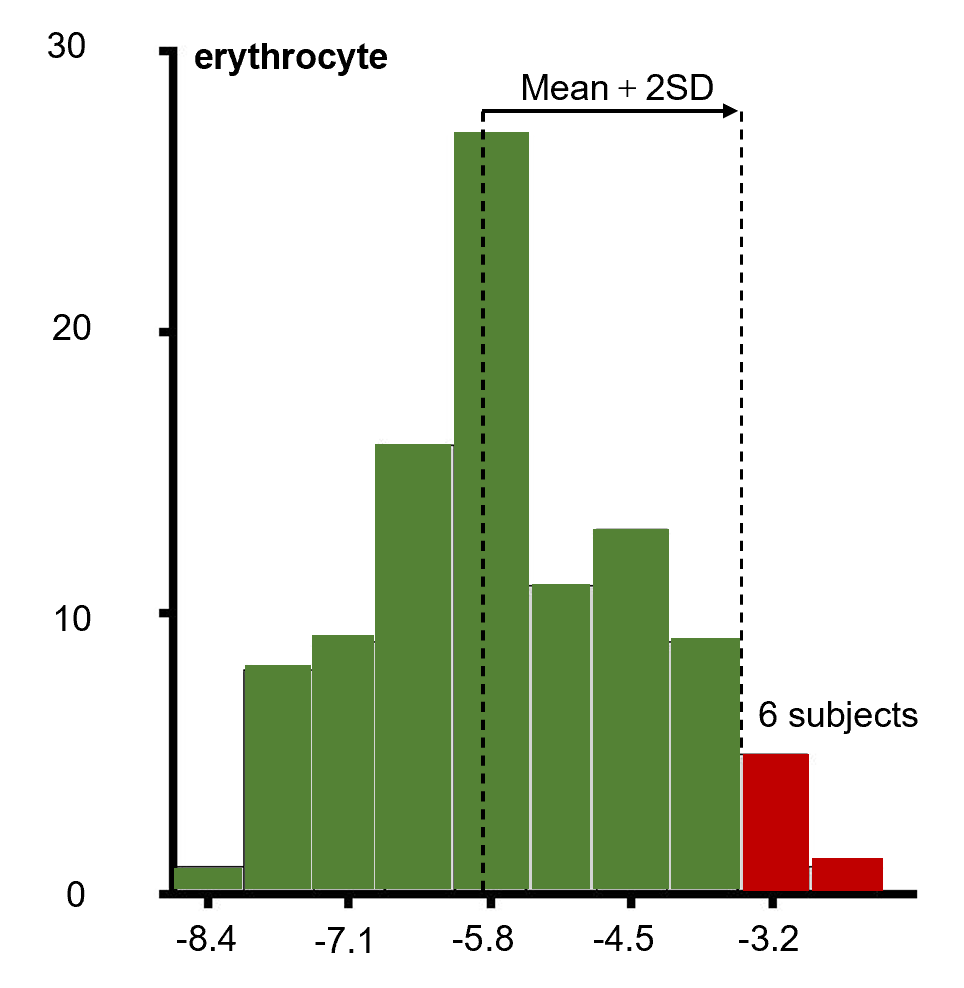


**Fig S4 a-b.** The validation of ferroptosis and lipid metabolism associated DEPs using IHC in an independent cohort. A, Representative images show the immunohistochemical staining of TFR1, TF, AIFM2, DPP4, and GCLC proteins in stable and unstable plaques in the plaque fibrous cap region. B, Immunohistochemical staining for SLC1A5, BID, and APOA5 proteins in the plaque lipid core region.


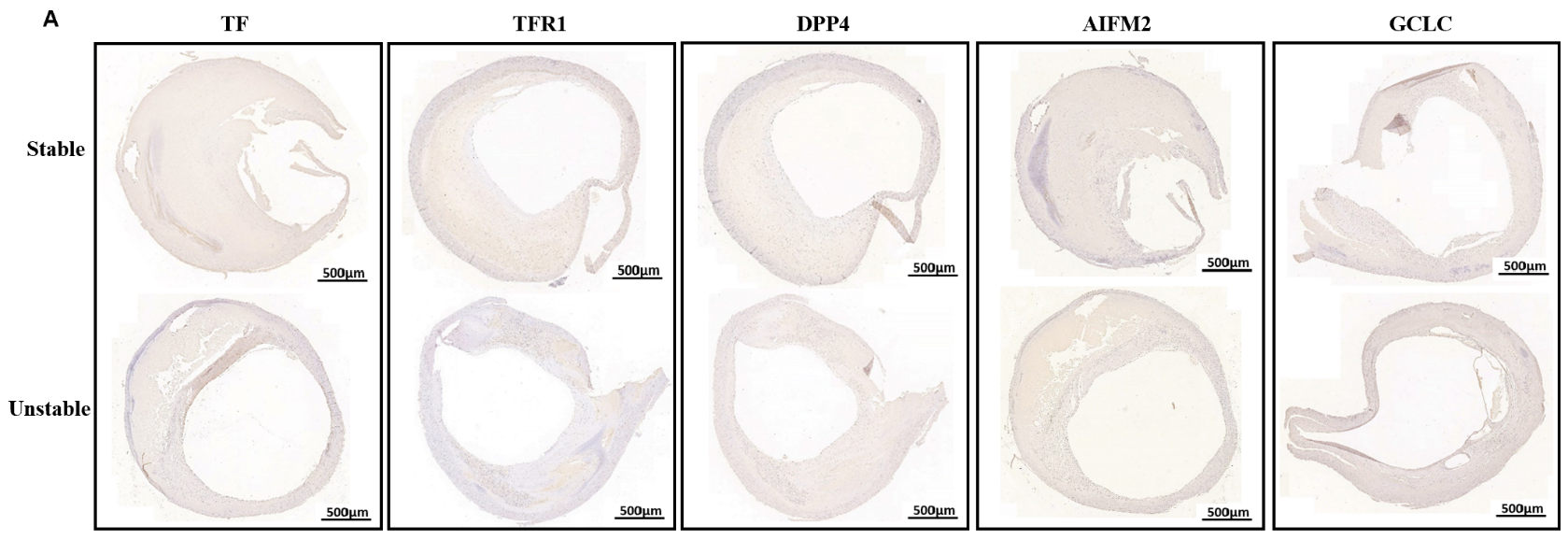


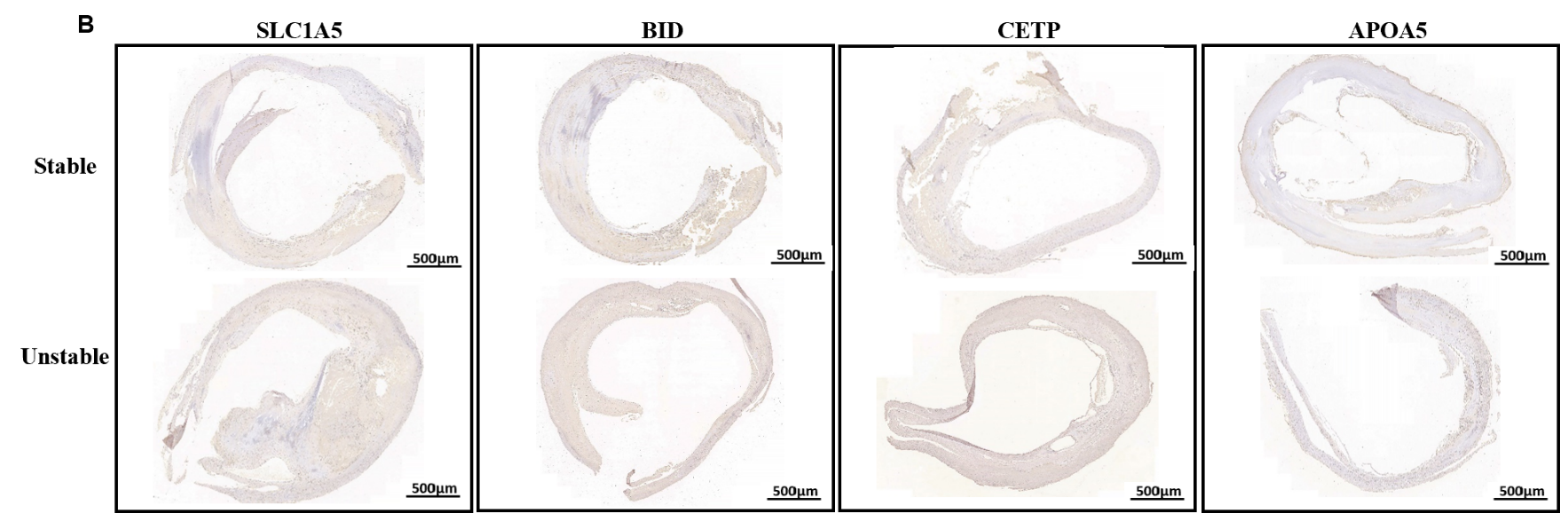


**
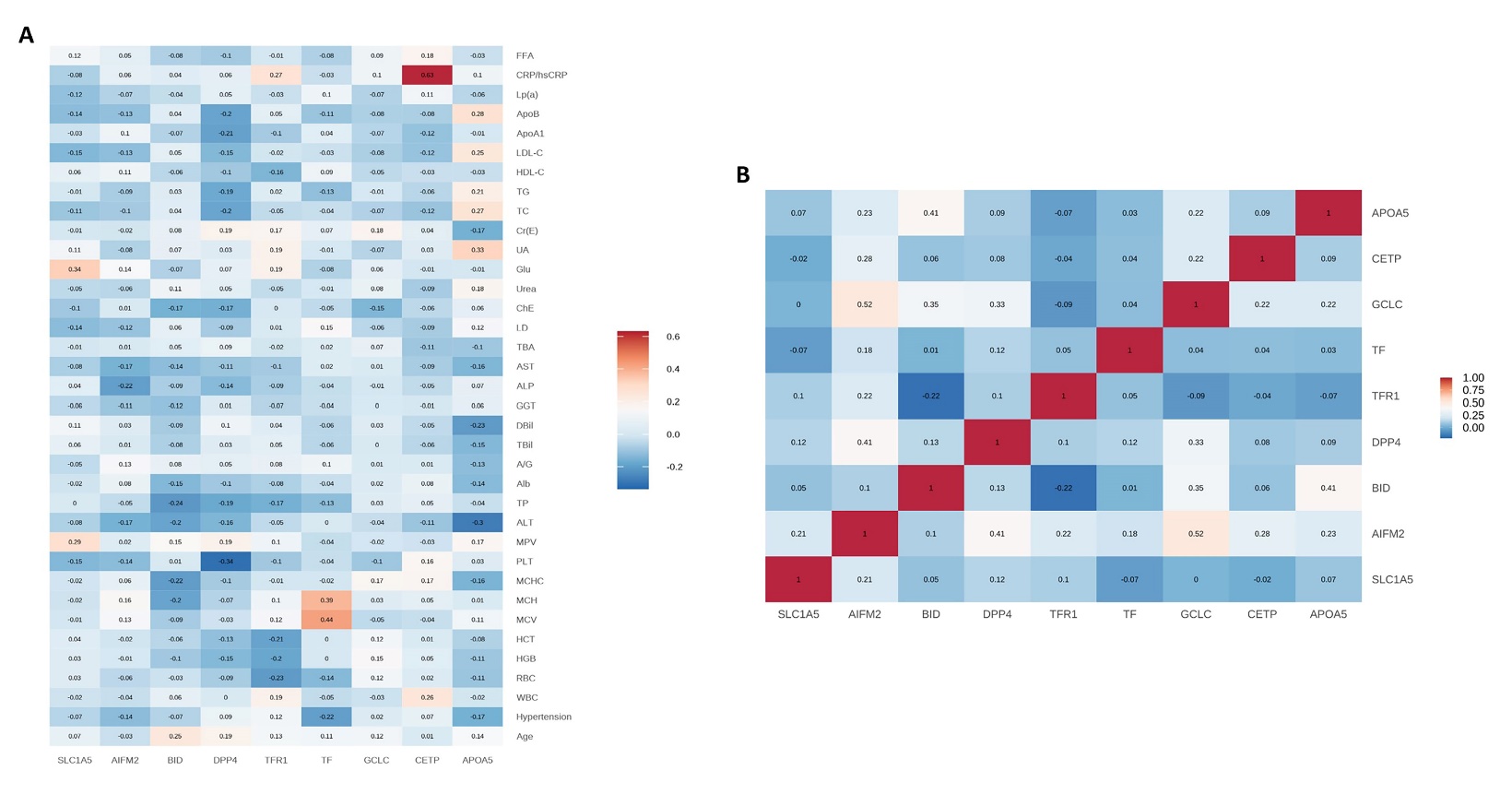
Fig S5 a-b**. Pearson correlation analysis of DEPs and clinical characteristics. A, The heatmap of the relationship between validated DEPs and clinical characteristics. B, The heatmap of the inner relationship of validated DEPs. DEPs, differentially expressed proteins. DEPs, differentially expressed proteins.
